# Micropeptides in the oncological dark matter: decoding their roles in tumor progression and therapy resistance

**DOI:** 10.1186/s12967-025-07310-8

**Published:** 2025-11-25

**Authors:** Qiqi Zhang, Haian Yang, Chunhua Wang, Diabate Ousmane, Jie Liu, Ziyu Liu, Wangrui Liu, Yuanliang Yan, Zhijie Xu, Junpu Wang

**Affiliations:** 1 Department of Pathology, Xiangya School of Basic Medical Sciences, Central South University, Changsha, China; 2 Department of Pathology, Xiangya Hospital, Central South University, Changsha, Hunan 410008 China; 3 Department of Pharmacy, Xiangya Hospital, Central South University, 87 Xiangya Road, Changsha, Hunan 410008 China; 4 Ultrapathology (Biomedical Electron Microscopy) Center, Department of Pathology, Xiangya Hospital, Central South University, Changsha, China; 5 Xiangjiang Laboratory, Changsha, China; 6 FuRong Laboratory, Changsha, China; 7 National Clinical Research Center for Geriatric Disorders, Xiangya Hospital, Central South University, Changsha, China; 8 Department of Oral Bioscience, Tokushima University Graduate School of Biomedical Sciences, Tokushima, Japan; 9 Department of Thoracic Surgery, Renji Hospital, School of Medicine, Shanghai Jiao Tong University, Shanghai, 200127 China

**Keywords:** Micropeptides, NcRNA, Tumorigenesis, Targeted therapy

## Abstract

Micropeptides are small peptide chains translated from non-coding RNAs (*ncRNAs*), typically ranging from < 100 to ~ 200 amino acids in length, and usually encoded by short or small open reading frames (*sORFs/smORFs*). Recent advances in high-throughput sequencing and multi-omics technologies have enabled genome-wide identification of *sORFs* across diverse species. Notably, a subset of these *sORFs* encodes functional micropeptides that play critical roles in biological processes such as inflammation, metabolism, and tumorigenesis. Emerging evidence suggests that tumor-associated micropeptides regulate key hallmarks of cancer, including proliferation, metastasis, metabolic reprogramming, angiogenesis, ion homeostasis, and immune evasion. In this review, we define the definition of micropeptides and discuss cutting-edge methodologies for their discovery, such as ribosome profiling (Ribo-seq), mass spectrometry (MS), and *sORF*-centric bioinformatics pipelines. Furthermore, we systematically summarize the functional mechanisms of micropeptides in tumor initiation, progression, therapeutic response, and drug resistance. This synthesis aims to provide novel perspectives on cancer biology and highlights micropeptides promising candidates for use as diagnostic biomarkers and targeted therapies.

## Introduction

Micropeptides are small peptide chains translated from open reading frames (ORFs) hidden in various transcripts, containing a start codon, fewer than 100 consecutive codons, and a stop codon, is classified as *sORF/smORF* [1–3]. Micropeptides play a vital regulatory role in various physiological and pathological processes. Peptides translated from sORFs/smORFs are called micropeptides [4, 5]. However, micropeptide biogenesis involves complex regulatory mechanisms beyond simple translation. Studies have shown that functional sORFs typically exhibit the following characteristics: (1) they are located at the 5’ end with a Kozak sequence or similar translation-enhancing elements [6]; (2) they may utilize internal ribosome entry sites (IRES) [7]; (3) they are associated with mature mRNA transcripts and can be detected as ribosome-protected fragments by Ribo-seq technology [8]. These peptide encoded by sORFs/smORFs have been shown to contribute to tumorigenesis, but their pathological mechanisms are still largely unclear due to limitations in sORF identification and translation detection techniques [9]. In recent years, with the rapid development of technologies such as high-throughput sequencing, MS analysis, and bioinformatics, the discovery and research of micropeptides have received strong support. Studies have shown that micropeptides play an important role in biological processes such as inflammatory responses and metabolic regulation [10–14]. Micropeptides act via: (1) acting as signaling molecules to interact with membrane receptors [15]; (2) regulating the activity or stability of key proteins (e.g., through competitive binding) [7]; and (3) influencing the functions of organelles such the mitochondrial oxidative phosphorylation [16]. However, due to the low translation efficiency of sORFs, the short half-life of micropeptides, and the oversight in traditional annotation pipelines, their pathological mechanisms still require further exploration.

Tumors are multifaceted abnormalities such as gene mutations, epigenetic changes and metabolic disorders in cells under the action of internal and external tumorigenic factors, resulting in uncontrolled cell proliferation, impaired differentiation, and abnormal tissue masses formed through clonal proliferation [17]. The biological behavior of tumors includes the ability to grow, invade, and metastasize autonomously [18], and can be further divided into benign tumors and malignant tumors. Tumors not only cause great suffering to patients, but also impose substantial economic burdens [19, 20]. The high cost of cancer treatment and the high mortality and disability rates seriously affect people’s health, leading to a decline in productivity and having a profound negative impact on socio-economic development [21]. Therefore, tumor management is a complex and multifaceted field that involves various strategies aimed at improving patient outcomes. Future anti-tumor therapies will likely focus on personalized medicine, leveraging genetic and molecular profiling of tumors to tailor treatments. Therefore, it is crucial to conduct in-depth research on the mechanisms of tumor development and find new therapeutic targets. Micropeptides, as an emerging research object, show great potential in tumor research” can be added. Emerging studies have shown that micropeptides are attractive biological factors that contribute to cancer pathogenesis [12]. The first *sORF* to be described as carcinogenic is the Cancer-Associated Small Integral Membrane Open reading frame 1 (*CASIMO1*). Micropeptide *CASIMO1* translated by long non-coding RNA (*lncRNA*) is overexpressed in hormone receptor-positive breast tumors, and when they are silenced, decreased proliferation is observed in various breast cancer (BC) cell lines. *CASIMO1* interacts with the BC oncogene squalene epoxidase (*SQLE*) to regulate cellular lipid homeostasis, thereby regulating the occurrence of BC [22]. Another microprotein is thought to be a positive regulator of the hippo-Ya common pathway in colon cancer. *CircPPP1R12A-73aa*, a microprotein encoded by the most abundant circRNA in colon cancer (CircPPP1R12A), promotes proliferation when overexpression leads to increased cell proliferation [23]. *CircPPP1R12A-73aa* induced transcriptional upregulation of components of the Hippo-yap pathway and increased Yes-associated protein 1 (YAP1) protein levels, suggesting that *CircPPP1R12A-73aa* may be an activator of the Hippo pathway [23]. Some identified microproteins, for example, non-annotated P-body dissociating polypeptide (*NoBody*) translated from *LINC01420/LOC550643* in leukemia and BC cells [24], they regulate fundamental processes that affect cancer cells, such as mRNA decay [24, 25]. Thus, the roles of this microprotein (and many others) as oncogenes or tumor suppressors may be highly tumor-specific and dependent on the cellular environment.

This review provides a comprehensive overview of the burgeoning significance of micropeptides encoded by *lncRNAs* in tumor biology and their implications for cancer treatment. It also examines current challenges and future directions highlighting the necessity for sophisticated computational methods and experimental approaches to uncover and elucidate the functions of these micropeptides. In summary, the exploration of *lncRNA*-derived micropeptides offers a novel and promising pathway for advancing cancer therapies and deepening our insights into the mechanisms underlying tumor behavior.

## Methods for the detection of micropeptides

### Ribosome profiling

Ribo-Seq is a deep sequencing-based omics technology developed by Ingolia et al. in 2009 that reveals the translation of thousands of *sORFs* in human cell lines and tissues [26, 27]. This technique uses nucleases to degrade mRNA that is not protected by ribosomes, resulting in the isolates ribosome-protected fragments (RPFs, typically 22–35 nt) of actively translated mRNA [28]. By performing deep sequencing of these fragments, Ribo-Seq is able to provide a genome-wide snapshot of the mRNA being translated, revealing translation efficiency and ribosomal occupancy [29]. The core strength of Ribo-Seq is its ability to directly measure the translation efficiency of mRNA, distinguish between the coding regions (CDS) and non-coding regions, and identify new translation events. It has been used to identify potential micropeptides in an increasing number of organisms, and a large number of potential functional micropeptides have been identified in biological tissues and cells such as humans, mice, and zebrafish [30–32]. However, it relies solely on ribosome binding, which may generate false positives, as not all sORFs associated with ribosomes are actually translated [33]. To address false positives from single ribosome binding, researchers have developed poly-Ribo-Seq technology to further distinguish untranslated individual ribosome-mRNA complexes by isolating mRNA bound to multiple ribosomes [34]. This technique provides more concrete evidence for active translation. In addition, Poly-Ribo-Seq technology verifies the reliability of its results by comparing them to Peptimics data. For example, this technique increased the number of *smORFs* with evidence of translation in S2 cells by a factor of nearly 4, from 59 (Peptide Atlas) to 228 (Poly-Ribo-Seq), showing high detection sensitivity and specificity [34]. Passive voice. Suggest: ‘Advancements in ribosome analysis technology enabled the creation of databases including GWIPS-viz (https://gwips.ucc.ie) [35, 36], TISdb (https://tisdb.human.cornell.edu) [37], OpenProt (https://openprot.org) [38, 39], and RiboSeqDB (https://riboseq.org). These databases provide abundant resources and powerful analytical tools for the study of micropeptides, advancing the field of micropeptide research.

### Mass spectrometry

MS is an important tool for micropeptide analysis. MS identifies genetically encoded peptides by separating and detecting ions based on mass-to-charge ratio (m/z) [40]. Researchers first lyse and digest samples, typically using trypsin to fragment proteins into peptides. Subsequently, the samples were separated and detected by liquid chromatography tandem mass spectrometry (LC-MS/MS) to obtain a sample mass spectrum. Experimental spectra are compared to theoretical spectra from protein databases [41]. Theoretically, in this way, micropeptides encoded by *sORF* can be discovered, which may not have been annotated or documented in existing theoretical mass spectra. However, the reliability and stability of MS is affected by a variety of factors. Differences in the selection and preparation of MS samples, the low abundance of micropeptides, their short lifetimes, and their high tissue specificity may interfere with the results [42]. Optimized enrichment methods are essential to verify SEPs. In recent years, researchers have developed a variety of enrichment and extraction methods to improve the detection of micropeptides [43]. Jiao Ma et al. found that acid precipitation and reversed-phase solid-phase extraction could better enrich micropeptides and significantly improve the recovery of trace proteins [44]. In addition, the high-resolution isoelectric focusing (Hi-RIEF) pre-sorting separation technique improves the detection of micropeptides by MS in a highly reproducible manner [45]. Electrostatic-hydrophilic interaction chromatography (ERLIC) allows charge-driven orthogonal separation of peptides prior to MS [46]. These methods work together to improve the detection of micropeptides by MS.

### Bioinformatics-based technology

Bioinformatics methods analyze genomic and transcriptome data through computational tools and databases to predict the coding potential of micropeptides, with the advantages of low cost, speed, and suitability for large-scale data processing. The FuncPEP database includes micropeptides up to 100 amino acids in length, and the existence and physiological function of these micropeptides have been confirmed by a variety of experimental methods such as Western Blot, MS, Ribo-Seq, Poly-Ribo-Seq, etc. FuncPEP not only provides researchers with a comprehensive micropeptide information resource, but also allows users to retrieve detailed information such as peptide name, *ncRNA*, peptide length, size, molecular weight, sequence, and function [47]. In addition, bioinformatics methods fill the gaps in traditional gene annotation by developing specialized tools. For example, MiPelid is a machine learning-based micropeptide identification tool that focuses on predicting the coding potential of *smORFs*. By analyzing the sequence information of the ORF, the tool is able to predict with 96% accuracy whether it encodes a micropeptide and correctly classify newly discovered micropeptides that are not included in the training or blinded data. MiPepid’s training dataset focuses on *smORFs* and excludes “regular-size” ORFs, significantly improving the accuracy of predictions [48]. Bioinformatics tools also include the RiboProfiling software package, which enables quality assessment and analysis of Ribo-Seq data, providing a complete flow from BAM file to codon coverage analysis [49]. However, the limitation of bioinformatics methods is that they may be limited by data quality and algorithm accuracy, and they need to be validated in combination with experimental methods such as MS and Ribo-Seq to improve the accuracy and efficiency of micropeptide detection and further explore the functions and applications of more micropeptides in tumors (Table 1).

**Table 1:** Comparison of the advantages and disadvantages of micropeptide identification techniques

Techniques	Advantages	Disadvantages	Reference
Ribo-Seq	Analyze translations at the genome-wide level Identify the translation start site	There are problems such as sequencing errors, RNA fragment contamination, and non-translated signals, which can introduce a large number of false positives	[29, 33]
Poly-Ribo-Seq	Focusing on mRNA on polysomes, it is able to reflect the dynamics of active translation	The demand for sample size is large, and the experimental cost is high Data interpretation relies on complex computational models	[34]
MS	High sensitivity for the detection of low-abundance micropeptides It can be used for large-scale protein identification and quantification.	Limited detection capacity for low-abundance proteins Complex sample handling and purification steps are required Data analysis is complex and requires a combination of multiple algorithms and databases	[40, 42, 43]
Bioinformatics-based technology	It can be combined with a variety of omics data for comprehensive analysis Ability to predict potential translational events and micropeptide function	Detection of low-abundance micropeptides is limited There is a need for high-quality reference databases	[47, 48]

## The regularity roles of micropeptides in tumorigenesis

### Cell proliferation

**Fig. 1:**
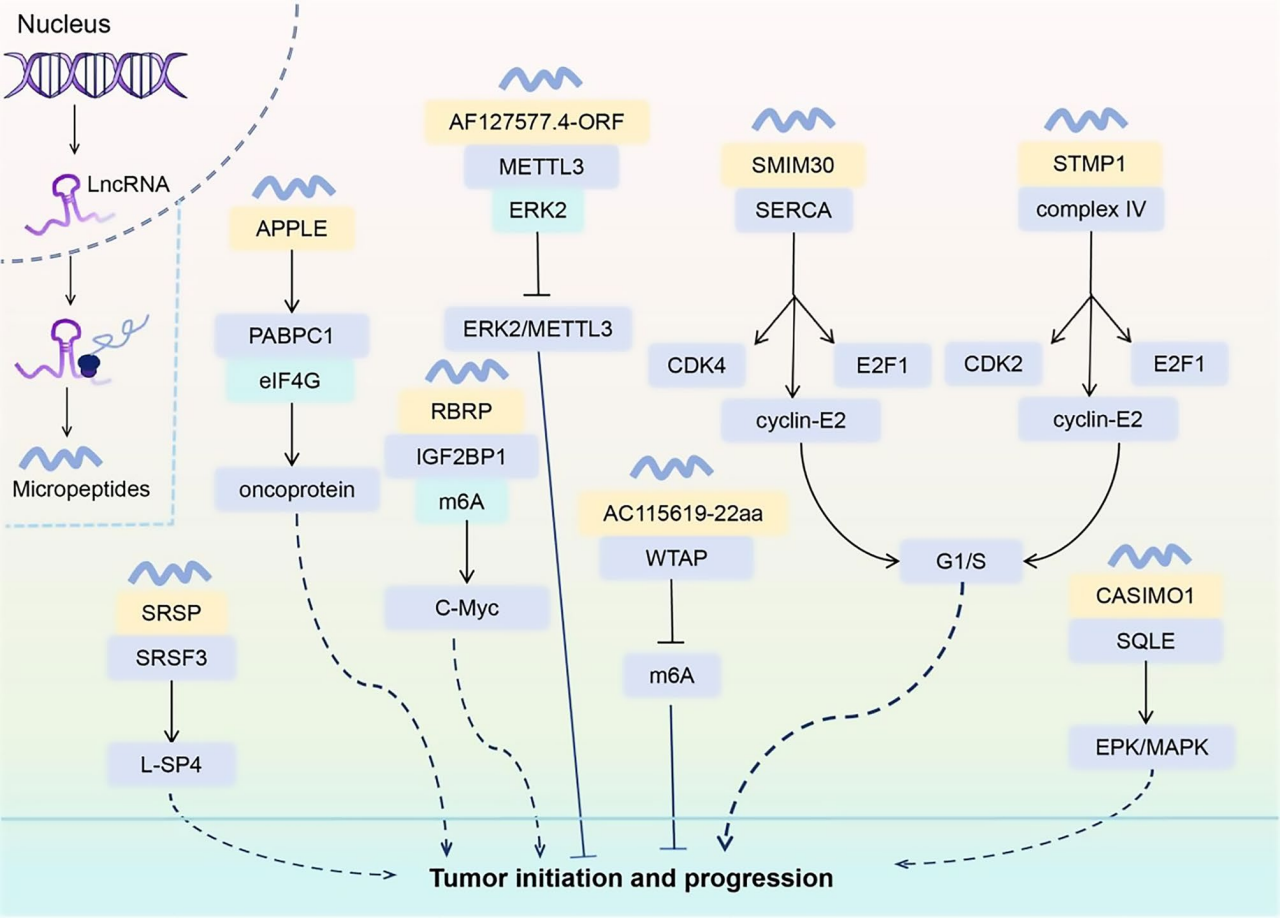
Mechanisms by which micropeptides regulate tumor cell proliferation. This figure summarizes the molecular mechanisms by which various micropeptides (e.g., SRSP, APPLE, RBRP, AF127577.4-ORF, AC115619-22aa, SMIM30, STMP1, CASIMO1) regulate tumor cell proliferation through transcriptional and translational control, m6A modification, cell cycle progression, and signaling pathways (e.g., ERK/MAPK)

While many roles are unclear, some micropeptides demonstrably affect proliferation. These micropeptides affect the occurrence and development of tumors by regulating key biological processes such as transcription and translation, RNA modification, cell cycle, and signaling pathways (Fig. 1).

By regulating the transcription and translation of cancer-associated molecules, the abnormality of micropeptides can demonstrably affect the occurrence and development of tumors. For example, Meng et al. found that the *LOC90024* encoded micropeptide SRSP interacts with serine/arginine-rich splicing factor 3 (SRSF3) to enhance its binding ability to transcription factor SP4 exon 3. This interaction promotes the production of an oncogenic long SP4 isoform (L-SP4) and drives colorectal cancer (CRC) progression [50] (Fig. 1). Sun and colleagues found that in acute myeloid leukemia (AML), the tumor microorganism APPLE, encoded by *ncRNA* transcripts, can enhance the efficiency of translation initiation complex formation, accelerate the conversion of mRNA to protein, and promote the development of hematopoietic malignancies by interacting with key factors of translation initiation [51] (Fig. 1).

N6-methyladenosine (m6A) modification affects RNA splicing, stability, translation, and degradation, and regulates RNA metabolism and gene expression through its “writer”, “eraser”, and “reader” proteins [52, 53]. Zhu et al. found that the micropeptide RBRP encoded by *LINC00266-1* in CRC can specifically bind to insulin-like growth factor-2 mRNA-binding protein 1 (IGF2BP1), alter the recognition and binding of m6A-modified mRNA by IGF2BP1. This interaction increases mRNA stability, translation efficiency, and promote tumorigenesis [54] (Fig. 1). On the contrary, in glioblastoma (GBM), Du’s group et al. found that the micropeptide AF127577.4-ORF, encoded by *lncRNA AF127577.4*, was able to specifically interact with methyltransferase-like protein 3 (METTL3) and mitogen-activated protein kinase 1 (MAPK1/ERK2). AF127577.4-ORF reduces METTL3 stability and m6A modification of mRNA by inhibiting ERK2-METTL3 interaction. The reduction of m6A modification levels affects the stability and translational efficiency of mRNAs of a series of oncogenes, and has strong antiproliferative functions in GBM [55] (Fig. 1). In addition, Zhang et al. in hepatocellular carcinoma (HCC) studies, found that *lncRNA AC115619* expressed at low levels encodes a micropeptide called AC115619-22aa. AC115619-22aa reduced overall m6A levels and inhibited tumor growth by binding to Wilms tumor 1-associated protein (WTAP) and hindering the assembly of the m6A methyltransferase complex [56] (Fig. 1).

Cell cycle dysregulation, especially defective G1/S transition, is a critical step in cancer development when it comes to regulating the tumor cell cycle. The findings from Yang’s report indicated that the micropeptide SMIM30, encoded by *LINC00998*, was upregulated in various malignancies and promoted tumor growth by reducing cytosolic calcium levels and promoting G1/S transition [57] (Fig. 1). In addition, Sang et al. demonstrated that the micropeptide STMP1 was overexpressed in HCC and enhanced G1/S transition by activating mitochondrial complex IV [58] (Fig. 1).

Micropeptides can also affect tumor cell proliferation by regulating kinase signaling pathways. Polycarpou-Schwarz et al. found that in hormone-dependent BC, *CASIMO1* interact with the oncogene *SQLE*, altering the activity or conformation of SQLE to activate the ERK/MAPK pathway [22] (Fig. 1). This pathway is composed of a series of protein kinases and transmits signals through a multistage phosphorylation cascade. Extracellular stimuli (e.g., growth factors) activate receptor tyrosine kinases, which in turn activate RAS, RAF, MEK, and ultimately phosphorylation of ERK. Phosphorylated ERK enters the nucleus and regulates the expression of genes associated with cell proliferation, thereby promoting BC cell proliferation [22].

### Energy metabolism

**Fig. 2:**
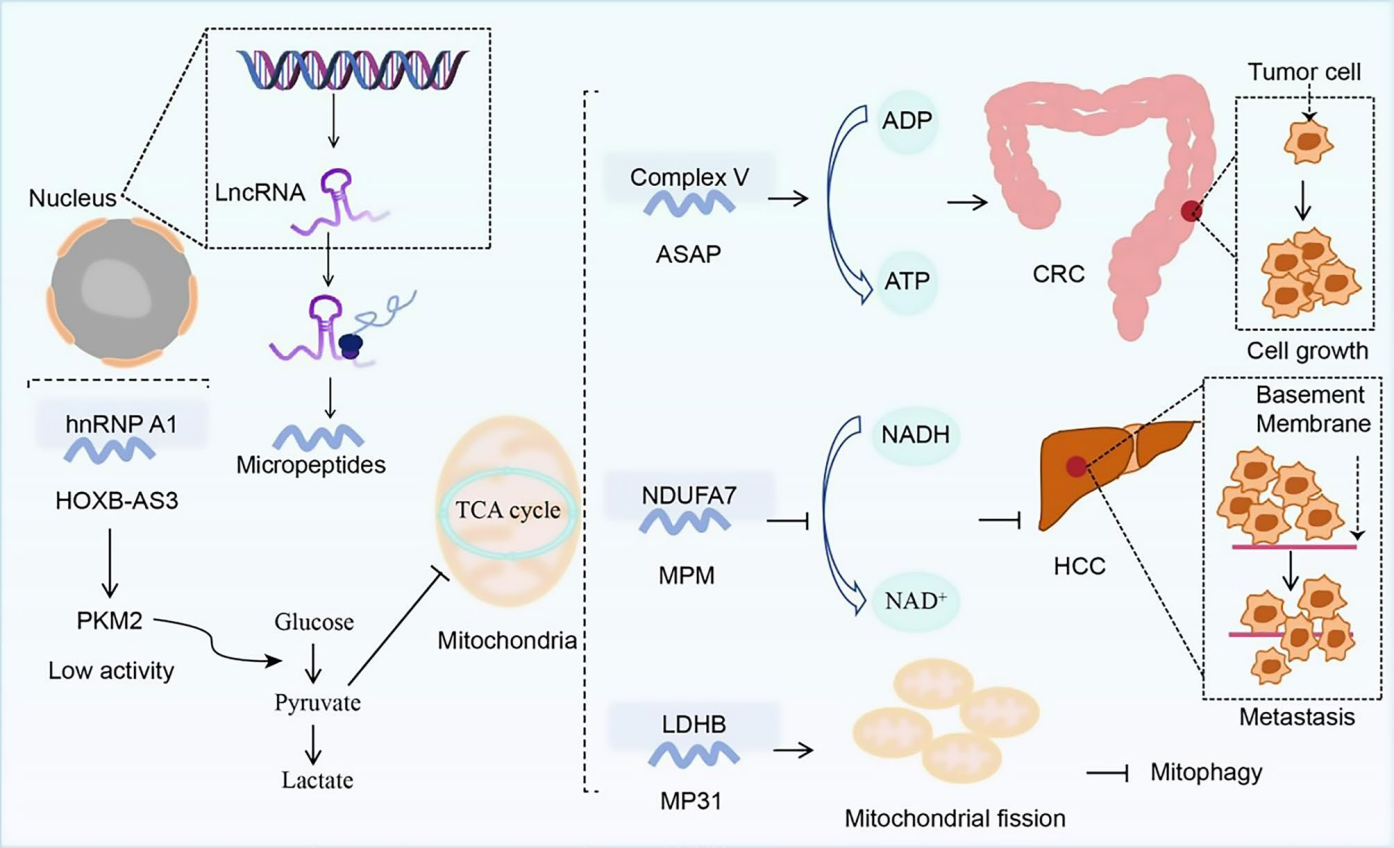
The role and mechanism of micropeptides in energy metabolism in tumor cells. The figure illustrates the localization and functions of micropeptides in the nucleus (e.g., HOXB-AS3) and mitochondria (e.g., ASAP, MPM, MP31): The HOXB-AS3 micropeptide inhibits glycolysis through the hnRNP A1-PKM2 axis; ASAP enhances ATP synthase activity to fuel CRC progression; MPM suppresses HCC metastasis by binding to NDUFA7 to reduce the NAD+/NADH ratio; MP31 induces mitochondrial fission while blocking mitochondrial flux

In addition, metabolic heterogeneity is intricately linked to tumor cell proliferation [59–61]. In terms of regulating tumor metabolism, Some micropeptides regulate tumor growth by modulating cellular metabolism. For example, the findings from Huang’s report indicated that the 53-aa length conserved peptide chain encoded by *lncRNA HOXB-AS3* in CRC, when bound to heterogeneous nuclear ribonucleoprotein A1 (hnRNPA1), antagonizes its mediated PKM splicing regulation, enables the formation of low-activity pyruvate kinase PKM2 isoform (Fig. 2), inhibits glucose metabolism reprogramming [62]. Ge and colleagues found that the mitochondrial localization micropeptide ASAP (ATP Synthase-Associated Peptide), encoded by *lncRNA LINC00467*, acts as an ATP synthase regulator and interacts with ATP synthase to promote mitochondrial ATP production, while increasing cellular oxygen consumption rate (OCR) and providing energy for the occurrence of CRC [63] (Fig. 2). MPM (Mitochondrial Micropeptide), also known as MOXI/MTLN, is a conserved polypeptide that contains 56 amino acids and exists in the inner membrane of mitochondria, playing an important role in mitochondrial respiratory activity [16, 64–66]. Xiao et al. discovered that MPM is downregulated in tumor tissue and represses metastasis by reducing mitochondrial complex I activity and the NAD+/NADH ratio via binding to NADH: ubiquinone oxidoreductase subunit A7 (NDUFA7) [67] (Fig. 2). Huang and his team revealed that a uORF-encoded microprotein, MP31, disrupts mitochondrial quality control (MQC) processes and inhibits tumorigenesis. Reexpression of MP31 in patient-derived GBM cells was found to induce loss of mitochondrial membrane potential (MMP) to trigger mitochondrial fission, but block mitochondrial flux (Fig. 2), resulting in the accumulation of damaged mitochondria in the cell, resulting in reactive oxygen species and DNA damage [68].

### Invasion and metastasis

Micropeptides alter tumor microenvironment (TME) by participating in complex signaling pathways within cells, thereby regulating tumor invasion and metastasis. In recent years, studies have shown that the role of micropeptides is twofold: on the one hand, some micropeptides can promote the migration and invasion of tumor cells; On the other hand, there are also micropeptides that have been found to inhibit this process. The specific effect depends on the type of micropeptide and the type of tumor.

**Fig. 3:**
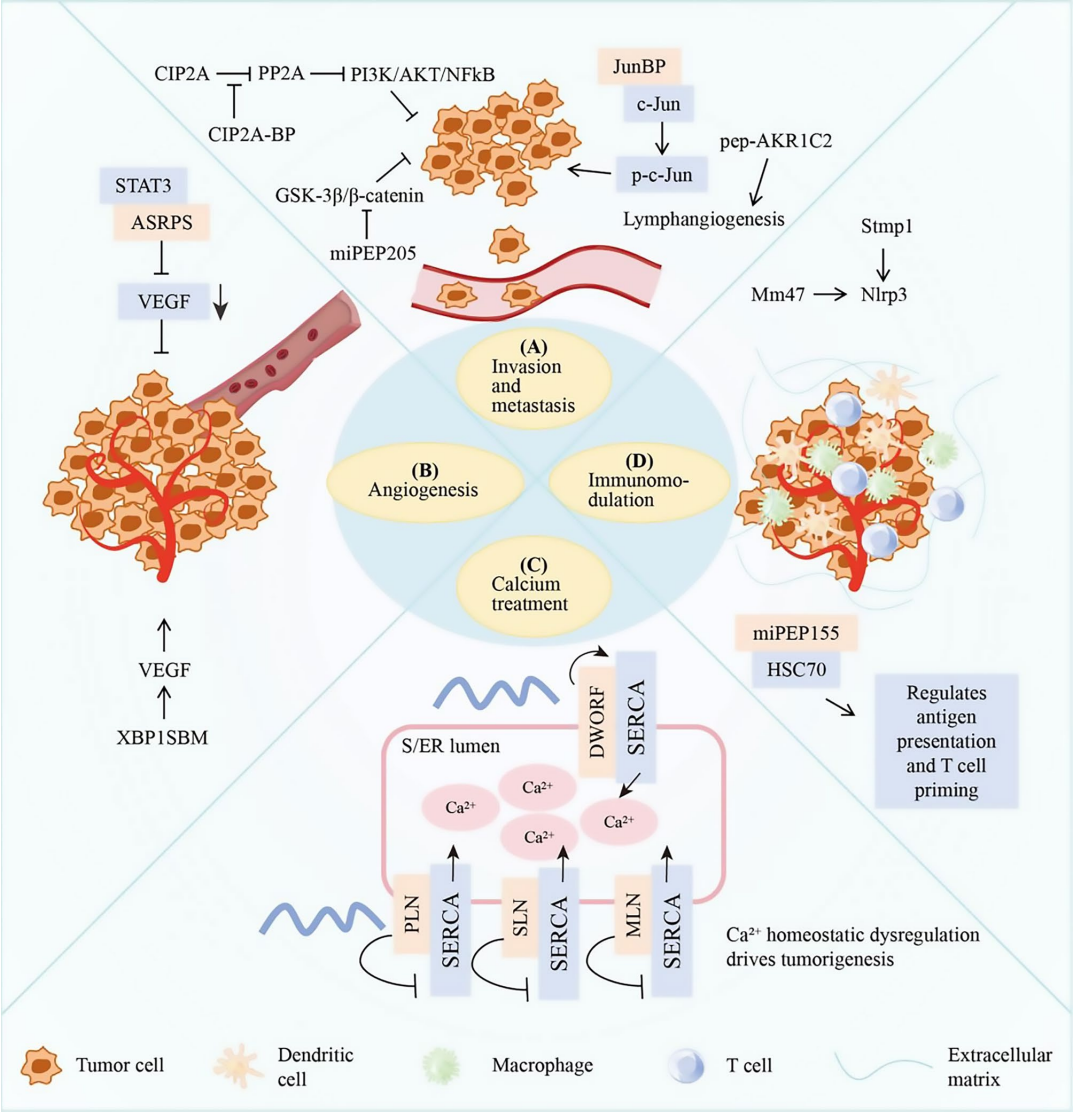
Micropeptides may regulate tumor cell migration and invasion, angiogenesis, ion homeostasis, and immune response. (**A**) Micropeptides (CIP2A-BP, miPEP205, JunBP, pep-AKR1C2) can regulate migration, invasion, related genes and signaling pathways, and affect the metastasis process. (**B**) Micropeptides (ASRPS, XBP1SBM) regulate tumor angiogenesis by regulating proangiogenic molecules, such as VEGF, and are involved in angiogenesis patterns. (**C**) Micropeptides (PLN, SLN, M LN, DWORF) can regulate Ca^2+^ levels, and their homeostatic dysregulation drives tumorigenesis and progression. (**D**) Micropeptides (miPEP155, Mm47, Stmp1) are able to regulate immune cell activation, antigen presentation, and inflammatory responses

In inhibiting tumor metastasis, the micropeptides CIP2A-BP and miPEP205 play an important role. CIP2A-BP is underexpressed in BC malignant cells, and its mechanism of action is closely related to the PI3K/AKT pathway [69]. Under normal physiological conditions, this pathway participates participates in various processes such as cell growth, metabolism, and survival. However, during tumorigenesis and development, this pathway is overactivated, resulting in abnormal proliferation, migration and invasion of tumor cells [70]. CIP2A-BP inhibits the PI3K/AKT pathway by competing with PP2A to bind CIP2A, thereby inhibiting BC metastasis (Fig. 3A). Notably, low levels of CIP2A-BP were found to be associated with lower overall survival in BC patients [69]. However, in HCC, overexpression of CIP2A-BP can promote the proliferation, invasion and metastasis of HCC cells [71]. This functional dichotomy may reflect tissue-specific interaction networks. In BC, CIP2A-BP functions mainly by competing with PP2A for CIP2A, while in liver cancer, there may be unknown targets that interact with CIP2A-BP, resulting in opposite functions. In addition, miPEP205 is a *lncRNA*-derived micropeptide. The mechanism of action involves the GSK-3β/β-catenin pathway [72]. GSK-3β is a serine/threonine kinase that phosphorylates β-catenin, degrades it ubiquitinately, and maintains low intracellular levels. When the pathway is abnormally activated, β-catenin accumulates into the nucleus, initiating the expression of genes related to cell migration and invasion, and promoting tumor development [73]. Mechanistically, EGR3 controls *lncRNA* MIR205HG and micropeptide expression, while miPEP205 promotes phosphorylation of GSK-3β at Tyr216. This cascade results in β-catenin degradation, inactivating the GSK-3β/β-catenin pathway and ultimately inhibiting the growth and metastasis of Triple-negative breast cancer (TNBC) [72] (Fig. 3A).

In promoting tumor metastasis, pep-AKR1C2, JunBP, and DLX6-AS1-encoded peptides have all been shown to be involved in this process [74–76]. pep-AKR1C2 is encoded by gastric cancer (GC)-derived exosomal *lncRNA (exo-lncAKR1C2)*. Studies have shown that pep-AKR1C2 enhances the tube formation and migration ability of lymphatic endothelial cells by regulating YAP phosphorylation and CPT1A expression, and promotes lymphatic vessel production and lymphatic metastasis in vivo [74] (Fig. 3A). Lymphangiogenesis provides a pathway for lymphatic metastasis of tumor cells, allowing them to spread through the lymphatic system to local lymph nodes and beyond [77–80]. In HCC, TGF-β transcriptionally activates LINC02551, encoding the 174-aa peptide JunBP, and the resulting JunBP binds to c-Jun and promotes its phosphorylation activation, thereby promoting HCC metastasis (Fig. 3A), and the activated c-Jun will also recruit more transcription factor SMAD3 to the promoter region of the *LINC02551*, forming a positive feedback to promote metastasis [75]. The short peptide encoded by the ORF of *lncRNA DLX6-AS1* also plays an important role in non-small cell lung cancer (NSCLC). The exogenously overexpressed *DLX6-AS1*-encoded peptide can significantly promote the proliferation, migration and invasion of NSCLC cells by activating the Wnt/β-catenin signaling pathway [76]. The Wnt/β-Catenin signaling pathway plays a central role in both embryonic development and tumorigenesis, and aberrant activation leads to the accumulation of β-catenin in cells, initiating the expression of related genes and promoting the malignant behavior of tumor cells [81–84].

Comparing the functions of these micropeptides, from the perspective of tissue types, the gene expression profiles and signaling pathways of different tumor tissues were different, for example, *CIP2A-BP* had opposite functions in BC and HCC [69, 71], reflecting the effect of tissue type on the function of micropeptides. In terms of signaling pathway interaction, *miPEP205* and *DLX6-AS1* encoded peptides acted on GSK-3β/β-catenin and wnt/β-catenin pathways, respectively, but produced opposite effects [72, 76], indicating that the same signaling pathway had different functions under the regulation of different micropeptides. In addition, post-translational modifications may also affect the function of micropeptides, although not explicitly mentioned in the current article, in other studies, post-translational modifications can change the stability and localization of proteins [85, 86]. Therefore, the diversity and complexity of micropeptide functions are closely related to tissue types, signaling pathway interactions, and possible post-translational modifications, and in-depth study of these factors can help to understand the mechanisms of tumor invasion and metastasis more comprehensively, and provide new targets and strategies for tumor treatment.

### Angiogenesis

Angiogenesis is a critical process for tumor growth, invasion, and metastasis [87–89]. When the tumor grows to a volume of 1–2 mm^3^, a microenvironment of hypoxia, abnormal vasculature, acidosis, and high interstitial pressure is gradually formed in the tumor tissue, which releases abundant growth factors and cytokines, stimulates angiogenesis and lymphatic genesis, and meets the needs of tumor growth and metabolism [87, 90]. This process is primarily driven by the signaling pathway mediated by vascular endothelial growth factor (VEGF) and its receptor (VEGFR) [91–94]. In recent years, studies have found that micropeptides play an important role in tumor angiogenesis, and they regulate the VEGF-VEGFR signaling pathway through multiple mechanisms, which in turn affect tumor growth and metastasis. For example, Wang’s group identified ERα-regulated *lncRNA LINC00908*, which is downregulated in TNBC and is associated with increased tumor growth and poor overall survival (OS). *LINC00908* encoded peptide ASRPS, a small regulatory peptide of STAT3, regulates angiogenesis in TNBC via the STAT3/VEGF pathway [95] (Fig. 3B). In addition, Wu et al. found that the survival-associated micropeptide XBP1SBM of 21 amino acids encoded by *lncRNA MLLT4-AS1* was upregulated in TNBC tissues and Gln-deprived TNBC cell lines, and its expression was promoted by Gln-deprivation-induced XBP1s transcription, which in turn retains XBP1s in the nucleus to enhance VEGF expression and promote angiogenesis and metastasis of TNBC [96] (Fig. 3B; Table 2). Angiogenesis enables tumor tissues to establish their own blood circulation system, providing sufficient nutrients for the growth of tumor cells, and also creating favorable conditions for the spread of tumor cells [97–99].

**Table 2:** Function of ncRNA-encoded micropeptides in tumors

Classify	NcRNA	Micropeptide	Size	Cancer types	Cell lines	Function	References
Cell proliferation	LOC90024	SRSP	130 aa	CRC	SW480, SW620, and HCT-116	Interact with SRSF3 to promote tumorigenesis and progression of CRC	[50]
	ASH1L-AS1	APPLE	90aa	AML	MOLM-13, NB4, THP-1, HL-60, MV4-11, ML-2	Promote hematopoietic malignancies by enhancing translational priming	[51]
	LINC00266-1	RBRP	71aa	CRC	SW480, SW620, HCT-116, MBA-MD-231, SK-OV-3, OVAR-3, HeLa	Promote the proliferation and colony formation of tumor cells	[54]
	lncRNA AF127577.4	AF127577.4-ORF	29aa	GBM	LN229 and U251	Modulation of ERK2/METTL3 interaction reduces GBM cell proliferation	[55]
	lncRNA AC115619	AC115619-22aa	22aa	HCC	——	Reduces m6A levels and reduces the growth of HCC	[56]
	LINC00998	SMIM30	59aa	HCC	Hela, HepG2 and SK-HEP‐1	Promotes G1/S transformation and cell proliferation	[57]
	lncRNA 1810058I24Rik	STMP1	47aa	HCC	HepG2, SK-Hep-1	Enhances mitochondrial complex IV activity to promote G1/S transformation and cell proliferation	[58]
	lncRNA	CASIMO1	10 kDa	BC	MCF7, KPL1, SKBR3, T47D, MDA-MB-231 and MCF10A	CASIMO1 interact with SQLE and affect cell cycle and proliferation	[22]
Energy metabolism	lncRNA	HOXB-AS3	53aa	CRC	HTC-116, MDA-MB-231, OVCAR-3, SK-OV-3	Inhibits the proliferation and tumor progression of cancer cells	[62]
	LINC00467	ASAP	94aa	CRC	LoVo, HCT116, RKO, SW480	Promotes mitochondrial ATP production, oxygen consumption rate, and cell proliferation	[63]
	LINC00116	MPM	56aa	HCC	SK-HEP-1, Hepa1-6, Hepa1c1c7, SNU-449, AML12	Decreased MPM expression promotes HCC transfer by increasing mitochondrial complex I activity and NAD/NADH ratio	[67]
	——	MP31	——	GBM	MES28, GSC23, HMC3, NHA	Disrupt the MQC process and inhibit the occurrence of GBM	[68]
Invasion and metastasis	LINC00665	CIP2A-BP	52aa	TNBC	MDA-MB-231, MCF-10 A, Hs578T	Inhibits migration and invasion of TNBC cells	[69]
	lncRNA	miPEP205	——	TNBC	MCF10A, MDA-MB-231, BT549	Inhibition of TNBC progression	[72]
	LINC02551	JunBP	174aa	HCC	HL7702, HepG2, Hep3B, ALEX, HLF, MHCC-97 H and HCC-LM3	JunBP facilitates HCC transfer by binding to c-Jun and subsequently promoting its phosphorylation activation	[75]
	lncAKR1C2	pep-AKR1C2	——	GC	HGC27 and MKN45	Modulation of YAP phosphorylation and CPT1A expression promotes LN transfer	[74]
Angiogenesis	LINC00908	ASRPS	60aa	TNBC	BT549, BT474, MCF7, T47D, MDA-MB-468、MDA-MB-231, HUVECs, Hs578T, SKBR3	Angiogenesis is regulated by the STAT3/VEGF pathway	[95]
	lncRNA MLLT4- AS1	XBP1SBM	21aa	TNBC	MDA-MB-231, Hs578T, HUVEC, MCF7 and T47D	Improves Gln levels and promotes angiogenesis	[96]

### Calcium (Ca2+) handling

Ion homeostasis, including calcium homeostasis, is indeed related to tumor characteristics and can be considered as a factor influencing tumor development and progression. Micropeptides may play an important role in the initiation and progression of cancer by regulating aspects of Ca²⁺ homeostasis, such as the activity of SERCA. Ca2 + homeostasis is essential for normal cellular function, especially in muscle cells [100, 101]. The contraction-relaxation cycle of skeletal muscle cells depends on the release of Ca2 + from the sarcoplasmic reticulum (SR) to the cytoplasm and the reuptake of Ca2 + into the SR, respectively [102, 103]. In addition, Ca²⁺ is important for maintaining mitochondrial homeostasis [102]. For example, Ca2 + overload can induce intrinsic apoptosis and even mitochondrial membrane depolarization [104–107]. In cancer, Ca²⁺ homeostasis is an important driver of tumorigenesis and progression [108–111]. Cancer cells exhibit cancer marker signatures by altering the expression and activity of Ca2 + regulators [112].

In recent years, it has been found that a variety of micropeptides are involved in the regulation of Ca²⁺ homeostasis by regulating SERCA activity. For example, phosphoproteins (PLN) and sarcospholipin (SLN) can bind SERCA and antagonize its function in Ca2 + treatment [113] (Fig. 3C). Another myoregulin (MLN), localized to SR, can inhibit the performance of SERCA. Structurally, it has a significant resemblance to PLN and SLN, with a transmembrane α-helix that can be docked in the grooves of SERCA. Functionally, it interacts with SERCA and hinders its pump activity [113] (Fig. 3C). In contrast to MLN, another DWORF can stimulate SERCA by interfering with the SERCA inhibitors PLN, SLN, and MLN [114]. DWORF is the only known endogenous peptide to activate SERCA (Fig. 3C). It is specifically expressed in heart and muscle cells and localizes to the SR membrane like MLN [115]. By regulating SERCA activity, these micropeptides not only affect the contractile function of muscle cells, but may also play a role in tumors by regulating Ca²⁺ homeostasis.

### Immunomodulation

In recent years, more and more studies have shown that micropeptides are able to modulate immune responses. This function is mainly reflected in its role in the activation of immune cells, cell signaling and antigen presentation. For example, Niu’s group discovered that human *lncRNA* encodes miPEP155 (P155), a 17-amino acid micropeptide, MIR155HG. In dendritic cells (DCs), Heat Shock 70 kDa Protein 8 (HSPA8/HSC70) acts as a chaperone protein that plays a key role in antigen trafficking and presentation. P155 can specifically bind to HSPA8 and effectively regulate the antigen presentation process mediated by major histocompatibility complex class II (MHC II) by interfering with the interaction between HSPA8 and heat shock protein 90 (HSP90), thereby affecting the activation of T cells and ultimately inhibiting autoinflammatory responses [116] (Fig. 3D). At the same time, Bhatta et al. identified a *lncRNA 1810058I24Rik* in mouse macrophages, which was downregulated in human and murine bone marrow cells exposed to lipopolysaccharide (LPS) and other Toll-like receptors (TLRs) and inflammatory cytokines, and encoded mitochondrial micropeptide-47 (Mm47) activates the NLR family pyrin domain-containing 3 (Nlrp3) inflammasome in macrophages and is involved in the body’s innate immune response and inflammatory processes [117] (Fig. 3D). Zheng and colleagues found that *lncRNA 1810058I24Rik* encodes the mitochondrial micropeptide Stmp1, which activates the Nlrp3 inflammasome pathway by regulating mitochondrial function and exacerbates microglia-mediated neuroinflammation [10] (Fig. 3D).

The regulatory effect of micropeptides on immune function suggests that they may play an unknown role in the formation and maintenance of tumor immunosuppressive microenvironment. Although there is no direct evidence to indicate the specific mechanism of action of micropeptides in the tumor immunosuppressive microenvironment, their potential role in immunomodulation suggests that they may have potential applications in tumor immunotherapy.

### Micropeptides and tumor treatment

#### Micropeptide-based tumor treatment strategies

It has been confirmed that inhibiting or enhancing the expression of certain micropeptides may even treat cancer by inducing apoptosis in cancer cells [118–120]. The method of inhibiting the proliferation, metastasis, invasion, or inducing apoptosis of cancer cells by increasing or decreasing the endogenous expression of certain micropeptides or directly injecting synthetic micropeptides into tumors to achieve therapeutic effects such as reducing the degree of cancer malignancy and prolonging the survival time of patients with advanced cancer (such as ovarian cancer) has become a new and effective treatment method in mouse experiments and some cancer models.

For example, Xu et al. discovered and identified a highly conserved 99 amino acid microprotein KRASIM in human and mouse cells, and found that KRASIM reduces KRAS protein levels by directly interacting with KRAS proteins in the cytoplasm. KRAS is a proto-oncogene that encodes a GTPase and can affect the proliferation, differentiation, and survival of tumor cells by regulating the RAF/MEK/ERK signaling pathway. Its downregulation can strongly inhibit the growth and proliferation of HCC cells. Therefore, the combination of KRASIM and KRAS can reduce its protein level, GTPase activity, and downstream ERK pathway, leading to inhibition of HCC cell proliferation [121]. In recent years, significant progress has been made in direct-targeting agents targeting KRAS, with the KRAS (G12C) inhibitors sotorasib and adagrasib achieving initial success in NSCLC and regulatory approval for both drugs [122, 123]. A large number of second-generation inhibitors are also being evaluated clinically. Among them, divarasib is 5 to 20 times more potent than sotorasib and adagrasib in preclinical studies [124]. In the future, KRASIM analogues are expected to be developed in combination with KRAS-targeted agents to enhance efficacy.

Li’s group discovered a new endogenous micropeptide MIAC encoded by *LncRNA RP11-469H8.6*, which can regulate the remodeling of SEP2/ITGB4 in the actin cytoskeleton by interacting with aquaporin 2 (AQP2) to inhibit the growth and metastasis of head and neck squamous cell carcinoma (HNSCC) [125]; Coincidentally, Mengwei Li et al., in renal cell carcinoma (RCC), the combination of MIAC and AQP2 can also inhibit the expression of EREG/EGFR, activate downstream pathways PI3K/AKT and MAPK, thereby inhibiting the proliferation (*P* < 0.01) and migration (*P* < 0.001) abilities of RCC cells, and even promote cell apoptosis (*P* < 0.001). Its effect is more effective than the current first-line treatment drugs, sunitinib and axitinib. In addition, the expression of MIAC in early patients is significantly higher than that in advanced patients, which can be used as an indicator for early diagnosis and prognosis of RCC patients [126].

Wu and colleagues discovered a Yin Yang 1-blocking polypeptide (YY1BM), which is encoded by *lncRNA LINC00278* on the Y chromosome and has a lower expression in esophageal squamous cell carcinoma (ESCC) than in adjacent normal tissues. YY1BM can render ESCC cells sensitive to apoptosis induced by nutritional deprivation (ND) by reducing the expression of eukaryotic elongation factor 2 kinase (eEF2K). In mouse experiments, intratumoral injection of exogenous YY1BM significantly improved the survival rate of male mice (*P* < 0.05) [127]. Intratumoral injection of YY1BM is expected to become one of the future treatments for ESCC (Table 3).

**Table 3:** Advantages of ncRNA-encoded micropeptide-related drugs in tumor therapy

NcRNA	Micropeptide	Cancer types	Function	drugs	Advantages	References
NCBP2-AS2	KRASIM	HCC	Interaction with KRAS protein inhibits oncogenic signaling of HCC	sotorasib adagrasib divarasib	Divarasib is 5 to 20 times more potent than sotorasib and adagrasib.	[121–124]
RP11-469 H8.6	MIAC	HNSCC	Interaction with AQP2 and inhibition of tumor growth and metastasis			[125]
AC025154.2	MIAC	RCC	Binds to AQP2 protein and inhibits RCC progression and metastasis	Chemically synthesized MIAC polypeptide	The antitumor effect at the dose of 15 mg/kg is better than that of the positive drugs sunitinib and axitinib	[126]
LINC00278	YY1BM	ESCC	Reduces the expression of eEF2K and sensitizes ESCC cells to ND-induced apoptosis	Exogenous YY1BM	In mouse experiments, intratumoral injection of exogenous YY1BM improved the survival rate of male mice	[127]

In the field of tumor treatment, micropeptide therapy has shown significant potential advantages. Although traditional chemotherapy can inhibit tumor growth to a certain extent, due to its lack of accurate identification of tumor cells, it will not only kill cancer cells, but also cause damage to normal cells, causing a variety of adverse reactions and affecting the quality of life of patients [128–130]. In stark contrast, micropeptide therapy has the significant advantages of high specificity and low toxicity [11, 131, 132]. Micropeptides can specifically act on tumor cell-related targets, reduce the impact on normal cells, and reduce the occurrence of adverse reactions. Therefore, slowing down or even terminating the progression of micropeptide-induced cancer may become one of the mainstream cancer treatment options in the future [9, 133, 134]. However, there are also some challenges associated with micropeptide therapy. Its low expression abundance, insufficient affinity with the target, susceptibility to protease-mediated degradation, and high difficulty in isolation and purification have hindered the clinical transformation process of micropeptide therapy [135, 136]. At present, most of the studies only stay at the stage of using certain micropeptides as potential targets for cancer therapy, and few studies have concluded that directly increasing or decreasing the expression of certain micropeptides can significantly inhibit cancer progression in mouse experiments, and the specific molecular pathways of micropeptide therapy have not been fully studied at this stage. Despite the above problems, the unique advantages of micropeptide therapy cannot be ignored. In the future, the combination of micropeptide therapy and traditional chemotherapy is expected to achieve complementary advantages, enhance the therapeutic effect, and effectively make up for the shortcomings of chemotherapy alone [137].

It has been mentioned that the use of yeast two-hybrid technology to screen and study intracellular proteins bound to micropeptides can improve the therapeutic mechanism of micropeptides [138]. We also found that some specific molecular pathways play an important role in peptide therapy, such as CIP2A bp inactivating the PI3K/AKT/ NFKB pathway to inhibit the invasion and metastasis of BC [69]; MIAC can activate PI3K/AKT and MAPK to inhibit the proliferation and migration of kidney cancer cells [126], suggesting that we can discover new micropeptides that affect tumor progression from the known molecular pathways affected by micropeptides, which may also be one of the hotspots for future research. Looking forward to the future, with the continuous deepening of research, the development of more types of peptide drugs will become an inevitable trend. These drugs include tumor homing peptides for targeted delivery of nanoparticles or EVs, peptide antagonists for cell surface proteins, and interfering peptides for protein-protein interactions (PPI) [139–141]. Through the research and development of these new peptide drugs, it is expected to solve or reduce the current shortcomings of micropeptides as therapeutic agents, and facilitate breakthroughs to the field of tumor treatment.

### Utilization of micropeptide-based strategies to overcome therapeutic resistance

At present, there are a few literatures mentioning the micropeptide in participating tumor therapeutic resistance, and its mechanism mainly links to DNA damage repair (DDR). Platinum-based drugs kill cancer cells by inducing DNA single-strand breaks (SSBs) and double-strand breaks (DSBs) [142]. However, cancer cells can repair damaged DNA through DDR mechanisms, leading to drug resistance [143–145]. Yu and colleagues identified a novel microprotein encoded by *lncRNA CTBP1-DT*, DDUP, which undergoes drastic conformational changes by activating ATR kinase-mediated phosphorylation, enhancing the interaction affinity with RAD18, thereby maintaining the aggregation of RAD18 at DNA damage sites. DDUP promotes DNA damage repair through RAD51C-mediated homologous recombination repair (HRR) and monoubiquitinated proliferative nuclear antigen (PCNA)-mediated damaged DNA replication repair (PRR) mechanisms, leading to cisplatin (CDDP) resistance [146]. The study by Ren et al. further showed that DDUP expression was significantly upregulated in CDDP resistant ovarian cancer cells. Patients with high expression of DDUP had shorter overall survival and disease-free survival after receiving platinum-based chemotherapy. The study also found that combination therapy with the ATR inhibitor Berzosertib could inhibit DDUP-mediated DDR maintenance and significantly enhance the sensitivity of ovarian cancer cells to CDDP [147]. In addition, Zhang’s group also discovered a micro peptide PACMP encoded by *lnc15.2* that directly regulates DDR, which can protect ctbp interacting protein (CtIP) from klhl15 mediated ubiquitination degradation. Poly (ADP ribose) (PAR) polymerase (PARP) inhibits the synthesis of lethality in cancer cells with homologous recombination (HR) defects, which is a necessary pathway for DNA double-strand break (DSB) repair. Therefore, PARP inhibitor (PARPI) therapy is highly effective therapeutic strategy. However, due to HR recovery, replication fork stabilization, or microhomology mediated terminal junction (MMEJ) addiction, PARPI treatment has a high resistance. The mechanism is that PACMP protects Ctlp, which can promote initial DSB terminal processing, HR, and MMEJ, leading to PARPi resistance [148]. At present, there are few studies on the drug resistance of tumors caused by micropeptides, which has a broad research prospect. Drug-induced apoptosis mainly focuses on inhibiting cell DDR to terminate cell processes, while the mechanism of tumor resistance caused by micro peptides focuses on promoting cell DDR processes and biological skills, bypassing the mechanism of apoptosis to achieve drug resistance. This suggests that when studying tumor resistance, we can discover new micro peptides that can cause tumor resistance by studying the DDR process.

### Summary and outlook

In the current field of research on inhibitory micropeptides, the existing detection techniques are still relatively limited. The low number of inhibitory micropeptides that have been identified has prompted the continuous exploration of new methods and advanced technologies to discover more functional micropeptides. In recent years, several studies have revealed the potential of micropeptides in regulating inflammatory responses and activating immune cells [11]. For example, P155 was able to modulate the antigen-presenting ability of DCs, inhibit the inflammatory response, and showed promising therapeutic effects in a mouse psoriasis-like model [116]. Since immune system activation can modulate the TME [149], an interesting hypothesis has emerged. Is it possible for these micropeptides to exert an inhibitive effect indirectly by coordinating a series of immune-mediated and microenvironment-related events? The study of He et al. provides strong support for this hypothesis. The immune agonist construct TACTIC developed by his research team is able to target multiple signaling pathways, remodel TME after radiotherapy, enhance the immunogenicity of tumor cells, and elicit a systemic immune response with memory effects [150].

These findings provide new ideas for the application of micropeptides in tumor treatment. Compared with traditional therapies, micropeptides have high specificity and activity, while exhibiting lower cytotoxicity and immunogenicity [11, 131, 132]. Traditional chemotherapy not only kills tumor cells, but also causes damage to normal cells, causing a variety of adverse reactions and drug resistance, which seriously affects the quality of life of patients [128]. In contrast, micropeptide therapy offers a novel approach with fewer side effects. These micropeptides can effectively target diseases and inhibit cancer cell growth and survival while sparing healthy cells [133], which is crucial for the clinical translation of micropeptide-based drugs. Currently, clinical trials based on micropeptides remain in the early stages. Research by Borbon et al. demonstrated that peptide receptor radionuclide therapy (PRRT) significantly improves survival rates in patients who underwent resection of gastroenteropancreatic neuroendocrine tumors, providing crucial insights for the clinical translation of micropeptides [151]. The successful application of PRRT not only demonstrates the potential of peptide drugs in tumor treatment, but also provides an important reference for the clinical translation of micropeptides. Of course, these micropeptides can also be used in combination with traditional anti-cancer drugs or radiotherapy and chemotherapy drugs to improve the effectiveness of cancer treatment [152].

However, micropeptide therapy also faces some challenges. The low expression abundance, short half-life, insufficient affinity with the target, and susceptibility to protease degradation of micropeptides have brought great difficulties to their therapeutic applications [135]. Currently, several strategies have been developed to address these challenges. In the development of cancer-targeted drugs, peptide chain modifications—including cyclization, glycosylation, esterification, amino acid sequence optimization, and polymer splicing—can be employed to refine the molecular structure of micropeptides. These modifications enhance membrane permeability, strengthen receptor binding affinity, improve metabolic stability, and reduce immunogenicity risks [9, 133]. In order to further break through the dilemma faced by micropeptide therapy, future research can be carried out from many aspects. On the one hand, CRISPR screening libraries can be developed to provide rich resources for in-depth study of micropeptide functions. For example, a large number of micropeptides associated with cell proliferation have been identified in gastric cancer samples using CRISPR screening technology, and more than 80% of the micropeptides exert cancer-promoting functions. In addition, this study also constructed a reference library of human micropeptide ORF, which provides important tools and data resources for micropeptide research [153]. On the other hand, targeted degradation technologies show great potential. Small molecule proteolysis-targeting chimeras (PROTACs) can specifically induce the degradation of bromodomain PHD finger transcription factors (BPTFs), thereby enhancing the anti-HCC ability of NK cells [154]. This technology not only specifically degrades cancer-promoting proteins, but also enhances the efficacy of immunotherapy by modulating the immune microenvironment. In addition, the combination therapy of immune checkpoint inhibitors and the optimization of delivery systems are also important research directions. The study found that the combination of SENP3 inhibitors and anti-PD-1 antibodies significantly enhanced the anti-tumor effect [155]. Micropeptides are potential immunomodulators and when used in combination with immune checkpoint inhibitors, they are expected to further enhance the efficacy of immunotherapy. In terms of optimizing the delivery system, EXO (TLR1/2 - STING) exosomes can efficiently activate anti-tumor immune responses, and have shown significant synergistic effects when combined with immune checkpoint therapies [156]. The use of exosomes as carriers to achieve targeted delivery by encapsulating micropeptides can enhance their enrichment and stability at tumor sites and open up new avenues for tumor treatment.

Overall, micropeptide therapy has shown significant efficacy in a variety of disease models and can effectively improve the survival rate of patients [118, 119]. However, most of the current studies on the function of micropeptides are based on cell models and mouse models, and lack validation in clinical or in vivo models [127]. Therefore, future research needs to further explore the regulatory mechanism of micropeptides, optimize the delivery system, and verify their safety and efficacy in humans through clinical trials.

## Conclusions

In recent years, the rapid advancement of multi-omics technologies and computational biology has provided compelling evidence that small open reading frames (sORFs) within ncRNAs can encode functional micropeptides. These micropeptides offer novel targets and strategic opportunities for cancer therapy. This review systematically elucidates the multifaceted mechanisms by which micropeptides regulate tumor proliferation, migration/invasion, angiogenesis, metabolism, ion homeostasis, and immunomodulation, while proposing innovative approaches to circumvent therapeutic resistance through micropeptide-based interventions. Nevertheless, only a limited fraction of ncRNA-encoded micropeptides have been experimentally validated to date. Through in-depth investigation of micropeptide regulatory networks and functions, coupled with cutting-edge technological platforms, we anticipate that micropeptides will realize their transformative potential in oncology—providing foundational resources for developing cancer biomarkers, identifying druggable targets, and engineering novel small-molecule peptide therapeutics, thereby heralding new hope for cancer patients.

## Data Availability

Not applicable’ for that section.
